# Effects of a Combined Physical Activity and Hypnosis-Based Mindfulness Intervention on ADHD Symptoms, Perceptual-Motor Abilities, and Balance in Children with Attention-Deficit/Hyperactivity Disorder

**DOI:** 10.3390/healthcare14131941

**Published:** 2026-07-01

**Authors:** Liza Jovičevič, Matej Majerič, Andrej Lapoša, Aleš Porčnik, Ivan Kneževič, Maks Tušak

**Affiliations:** 1 University Psychiatric Clinic Ljubljana, Chengdujska 45, 1000 Ljubljana, Slovenia; 2 Faculty of Sport, University of Ljubljana, Gortanova 22, 1000 Ljubljana, Slovenia; matej.majeric@fsp.uni-lj.si; 3 Department of Plastic, Reconstructive, Aesthetic Surgery and Burns, University Medical Centre Ljubljana, 1000 Ljubljana, Slovenia; andrej.laposa@kclj.si (A.L.); ales.porcnik@kclj.si (A.P.); 4 Department of Surgery, Faculty of Medicine, University of Ljubljana, 1000 Ljubljana, Slovenia; ivan.knezevic@kclj.si; 5 Transplantation Centre, University Medical Centre Ljubljana, 1000 Ljubljana, Slovenia; 6 Surgical Clinic, University Medical Centre Ljubljana, 1000 Ljubljana, Slovenia; maks.tusak@kclj.si

**Keywords:** ADHD, intervention, physical activity, hypnosis-based mindfulness, mindfulness, perceptual-motor abilities, balance

## Abstract

**Background**: Attention-Deficit/Hyperactivity Disorder (ADHD) is the most prevalent behavioral disorder in children. Physical activity (PA) and hypnosis-based mindfulness training (HBMT) have both been proposed as complementary approaches in the management of ADHD. The present study examined the effects of a combined PA and HBMT intervention on ADHD symptoms, perceptual-motor abilities, and balance in children with ADHD. **Methods**: The sample comprised 20 participants in the experimental group (EG) and 19 in the control group (CG). All participants were boys diagnosed with ADHD, aged 8–11 years. The intervention in the EG included PA and HBMT, whereas the CG received unsystematic education about emotions. The program was conducted twice weekly for 60 min over a period of three months. The severity of ADHD symptoms was assessed using the ASEBA TRF/6–18 questionnaires. Perceptual-motor abilities were evaluated using the Test of Perceptual-Motor Skills. Static balance was measured using a tensiometric force platform, and dynamic balance was assessed with the Biodex Balance System. For variables in which no statistically significant baseline differences between groups were observed, intervention effects were analyzed using a two-way repeated-measures analysis of variance (ANOVA). For variables showing statistically significant baseline differences, analysis of covariance (ANCOVA) was applied to examine the influence of initial intergroup differences on post-intervention outcomes, as well as on follow-up assessments conducted three and six months after the completion of the intervention. **Results**: Participants in the EG demonstrated significant short-term and long-term reductions in ADHD symptom severity. In addition, improvements were observed in perceptual-motor abilities and dynamic balance, while static balance improved immediately following the intervention. **Conclusions**: The findings suggest that a structured program combining physical activity and hypnosis-based mindfulness may represent a promising complementary intervention for children with ADHD. However, larger randomized controlled trials are needed to confirm these findings and further evaluate their long-term clinical relevance.

## 1. Introduction

### 1.1. Developmental and Behavioral Characteristics of Children with ADHD

Children with ADHD experience difficulties across multiple developmental domains, including attention, emotional regulation, motor functioning, cognition, and social interaction. Common challenges include impaired concentration, organizational difficulties, impulsivity, and problems with academic performance and peer relationships [1,2,3,4,5]. In addition, children with ADHD frequently exhibit clumsiness, poorer motor coordination, increased risk of injury, and deficits in both fine and gross motor skills [6,7,8].

ADHD may also affect emotional and social functioning. Difficulties with self-control, frustration tolerance, and interpretation of social cues can contribute to interpersonal conflicts and social exclusion [9,10]. Motor restlessness is particularly characteristic, with children often displaying excessive movement and difficulties remaining still even in situations requiring sustained attention [11]. These challenges frequently affect not only the child but also parents, teachers, and other caregivers, who may experience difficulties in managing behavior and providing effective support [12].

Overall, ADHD has a substantial impact on everyday functioning and may influence educational achievement, social participation, emotional well-being, and long-term developmental outcomes.

### 1.2. Motor Skills, Sensorimotor Development, and Executive Functions

Children with ADHD consistently demonstrate poorer motor performance than their peers, particularly in motor coordination, balance, reaction time, and movement control [13,14,15,16,17]. Studies estimate that 30–50% of children with ADHD experience clinically relevant motor coordination difficulties, and many also present with impairments affecting both fine and gross motor skills [15,17]. These difficulties have been linked to deficits in attention, executive functioning, and sensorimotor integration and may significantly affect everyday functioning [18,19,20,21].

Sensorimotor development plays an important role in early childhood because perception, movement, and cognitive functioning develop through continuous interaction with the environment [22,23]. Efficient processing and integration of sensory information support motor planning, coordination, balance, and adaptive behavior [24]. In children with ADHD, sensorimotor functioning may be compromised by difficulties in processing sensory input and body-position awareness, which can contribute to motor coordination problems and balance deficits [25].

Because sensorimotor functioning is closely connected to learning, emotional regulation, and social participation, it represents an important target for supportive interventions. These findings highlight the potential value of movement-based and sensory-rich programs that promote perceptual-motor experiences and functional development in children with ADHD.

In addition to motor and sensorimotor difficulties, impairments in executive functions represent another important feature of ADHD and contribute substantially to everyday functioning. Executive functions encompass cognitive processes such as attention, inhibition, working memory, and cognitive control, which are essential for goal-directed behavior [26,27,28,29,30]. Research indicates that many children with ADHD demonstrate deficits in these domains, including poorer inhibitory control, weaker working memory, and slower reaction times compared with their typically developing peers [31,32,33,34,35,36,37,38,39,40,41,42,43,44,45].

Executive functioning is also closely related to motor performance. Studies using dual-task paradigms have shown that children with ADHD may exhibit altered gait patterns and reduced performance when motor and cognitive demands are combined, suggesting difficulties in coordinating cognitive and motor processes simultaneously [27,28]. Because executive functions are closely linked to attention, learning, behavioral regulation, and everyday functioning, they represent an important target for intervention in children with ADHD [45,46].

Overall, the literature suggests that motor deficits, sensorimotor problems, and executive dysfunction should not be regarded as peripheral features of ADHD because they substantially influence school functioning, participation in physical activity, social integration, self-image, and long-term psychosocial adjustment. At the same time, the field remains characterized by heterogeneous samples and methods, indicating a need for more longitudinal and multidimensional studies.

### 1.3. Complementary Therapeutic Approaches

Alongside pharmacological and psychotherapeutic interventions, there is growing interest in complementary approaches for ADHD that aim to improve symptoms and overall functioning through nonpharmacological strategies. Commonly studied approaches include neurofeedback, physical activity, mindfulness, dietary modification, and nutritional supplementation [47,48,49,50]. Although some evidence suggests that these interventions may contribute to symptom reduction and improved self-regulation, findings remain heterogeneous and are often limited by methodological constraints, including small sample sizes, lack of blinding, and short follow-up periods [49,50]. Therefore, complementary approaches are generally recommended as part of a multimodal treatment plan rather than as standalone interventions.

Neurofeedback and biofeedback have also been investigated as complementary interventions for ADHD. Some studies suggest beneficial effects on attention and self-regulation, although findings remain mixed and methodological limitations persist [48]. Despite promising results, these approaches were not examined in the present study.

Physical Activity: Although physical activity is often associated mainly with bodily changes, it also exerts important effects on the brain and psychological well-being [51]. Research has shown that physical activity positively influences physical characteristics, metabolism, motor performance, and psychological functioning [52,53].

Several mechanisms may explain the beneficial effects of physical activity in ADHD. Ma [51] argued that appropriate exercise may also act therapeutically in conditions such as depression, anxiety, and ADHD. Ng et al. [49] suggested that physical activity, similarly to medication, may influence dopamine and norepinephrine levels, while Hillman et al. [54] added that exercise stimulates the release of serotonin and endogenous opioids, potentially improving mood and attention.

Empirical studies have reported beneficial effects of exercise programs on ADHD symptoms. Kang et al. [55] found reduced behavioral disturbances after a 6-week program of 90 min aerobic exercise twice weekly, Chou and Huang [56] reported improved attention after an 8-week program, and Fritz and O’Connor [57] observed reduced depression, anxiety, and fatigue after daily 20 min cycling.

Berwid and Halperin [58] emphasized improvements in executive functioning, while Pontifex et al. [59] and Piepmeier et al. [60] reported better attention, organization, and productivity.

Den Heijer et al. [61] described short-term physical improvements and more sustained improvements in attention and behavior, Arumugam and Parasher [52] noted short-term gains in endurance and longer-term enhancement of cognitive and social competencies, and Chan et al. [62] highlighted benefits for executive functions, motor skills, and ADHD symptoms.

Physical activity may also be especially important for children with ADHD because motor difficulties are common in this population. Structured movement tasks can target coordination, balance, postural control, visuomotor integration, and auditory-motor processing, all of which may affect school participation, play, peer interaction, and self-confidence. Programs that include fundamental movement patterns, balance exercises, dual-task activities, and games requiring attention to verbal instructions may therefore address both motor and cognitive aspects of ADHD. From this perspective, physical activity is not only a means of improving general fitness but also a structured learning environment in which children can practice attention, impulse control, self-regulation, cooperation, and adaptive responses to changing task demands.

Although research on physical activity and ADHD in children remains relatively limited, existing studies generally indicate positive effects on symptom reduction and suggest that both acute and structured exercise interventions may influence psychological and behavioral outcomes relevant to ADHD, despite differences in intervention duration, intensity, and type of exercise.

Overall, the literature supports the inclusion of physical activity in the comprehensive treatment of children with ADHD because both short- and longer-term programs may improve cognition, concentration, impulsivity, emotional stability, and sometimes family dynamics. Nevertheless, many studies are methodologically limited by small samples, insufficiently standardized exercise protocols, and inadequate control groups, and long-term effects remain less well established. More randomized controlled studies with larger samples and clearly defined activity protocols are therefore needed. Integrated programs that combine structured physical activity with attentional and self-regulatory components may be particularly valuable, as they may address the multidimensional nature of ADHD more comprehensively than interventions targeting only one domain.

Mindfulness: Mindfulness has also become increasingly common in work with children with ADHD and is generally understood as a form of self-regulation that helps individuals recognize, accept, and manage ADHD symptoms. In the context of ADHD, mindfulness is particularly relevant because it directly addresses several difficulties commonly observed in this population, including distractibility, impulsive responding, emotional reactivity, poor self-monitoring, and difficulties sustaining attention. Kabat-Zinn [63] described mindfulness as being cultivated through relaxed abdominal breathing, various forms of meditation involving observation of thoughts and emotions during rest, walking, or movement, and systematic muscle tension and relaxation. In this sense, mindfulness involves awareness of bodily sensations, breathing, thoughts, emotions, and the surrounding environment, while encouraging non-judgmental observation and reduced automatic reactivity.

This approach may be especially useful for children with ADHD because it teaches them to notice internal experiences before immediately reacting to them. Through repeated practice, children may learn to observe thoughts, emotions, bodily sensations, and impulses as transient experiences rather than as signals that require immediate behavioral action. Such training may support emotional regulation, impulse control, attentional stability, and a calmer relationship toward one’s own thoughts and feelings.

Although relatively few studies have examined mindfulness-based training in ADHD, and these studies share methodological limitations similar to those seen in exercise research, the available findings are encouraging. Haydicky et al. [64] and Chung Chan et al. [65] reported improvements in executive task performance, attention, working memory, and cognitive control. Behbahani et al. [66] found that parents perceived reductions in both children’s ADHD symptoms and their own psychological stress after mindfulness programs, while Chung Chan et al. [65] additionally emphasized better behavioral control. Lo et al. [67] reported reduced impulsivity and fewer uncontrolled bodily responses, such as tics. These findings suggest that mindfulness may influence not only the child’s attentional and behavioral regulation but also the wider family and support environment.

Taken together, the literature suggests that mindfulness may reduce ADHD symptoms and improve key cognitive functions negatively affected by the disorder, particularly attention, working memory, and behavioral control, while also benefiting parents and the wider support environment. However, most studies have used small samples, lacked long-term follow-up and appropriate control groups, or employed inconsistent mindfulness protocols, and outcomes have often relied heavily on parent ratings. More randomized controlled trials with clearly defined methods and longer follow-up are therefore needed before firm conclusions can be drawn about the long-term role of mindfulness in ADHD treatment.

In this study, the mindfulness component was adapted within a clinical hypnosis-based framework, which is described in detail in the Methods section.

Although mindfulness and clinical hypnosis are distinct approaches, comparative literature suggests that they partially overlap in many mechanisms such as focused/selective attention, absorption, relaxation, and increased imagery, while differing in the role of treatment method (detached observation vs. therapeutic suggestion), metacognitive monitoring (the ability to observe thoughts and emotions as transient mental events), general reality orientation (GRO) and perceived agency (the subjective sense of control) [68,69].

Guided imagery and relaxation techniques are frequently incorporated into both mindfulness- and hypnosis-based interventions. Relaxation is commonly used to facilitate focused attention and reduce physiological arousal, whereas guided imagery employs mental representations to support attention, self-regulation, and therapeutic goals. Although these techniques may be used within both approaches, they should be regarded as complementary methods rather than as synonymous with mindfulness or clinical hypnosis.

This conceptual overlap supports the development of integrative interventions in which mindfulness-based exercises are adapted within a clinical hypnosis-informed framework.

Although physical activity and mindfulness-based interventions have each shown promising effects in children with ADHD, most previous studies have examined these approaches separately. Physical activity primarily targets motor functioning, executive processes, attention, and behavioral regulation, whereas mindfulness-based approaches aim to strengthen self-awareness, attentional control, emotional regulation, and impulse management. The combination of these approaches may therefore provide complementary benefits by simultaneously addressing both motor and self-regulatory difficulties that are characteristic of ADHD. To date, however, evidence regarding the combined application of physical activity and hypnosis-based mindfulness in children with ADHD remains limited.

To our knowledge, no previous randomized controlled trial has investigated the effects of a structured intervention combining physical activity and hypnosis-based mindfulness on ADHD symptoms, perceptual-motor abilities, and balance in children with ADHD. The present study therefore aimed to examine both the short-term and long-term effects of such an intervention in a sample of boys diagnosed with ADHD.

## 2. Materials and Methods

The present study investigates whether a structured program combining physical activity and hypnosis-based mindfulness can reduce ADHD symptom severity and improve perceptual–motor abilities, static balance, and dynamic balance in children with ADHD aged 8–11 years.

**H_1_:**  *Children in the experimental group will demonstrate greater reductions in ADHD symptom severity at post-intervention and follow-up assessments compared with children in the control group.*

**H_2_:**  *Children in the experimental group will demonstrate greater improvements in perceptual–motor abilities at post-intervention and follow-up assessments compared with children in the control group.*

**H_3_:**  *Children in the experimental group will demonstrate greater improvements in static balance at post-intervention and follow-up assessments compared with children in the control group.*

**H_4_:**  *Children in the experimental group will demonstrate greater improvements in dynamic balance at post-intervention and follow-up assessments compared with children in the control group.*

Study Design and Participants: A randomized controlled trial was conducted involving children diagnosed with ADHD. Participants were randomly assigned in a 1:1 ratio to either an experimental group (EG) or an active control group (CG). Randomization was performed using sealed opaque envelopes prepared by an individual who was not involved in participant recruitment, intervention delivery, or outcome assessment. Each envelope contained a card indicating assignment to either Group 1 (experimental group) or Group 2 (active control group). Allocation was concealed until group assignment. Participants and their parents were not informed of the specific group allocation or study hypotheses during the intervention period.

A standardized measurement protocol was implemented at four time points: baseline (pre-intervention), immediately post-intervention, and at three- and six-month follow-ups. All participants were enrolled on a voluntary basis, with informed consent obtained from their parents or legal guardians. Ethical approval for the study was granted by the Ethics Committee of the Faculty of Sport (No. 033-14/2021-2) and the Doctoral Studies Committee of the University of Ljubljana (Decision 2.11., dated 7 June 2022).

A total of 43 children with a clinical diagnosis of ADHD were initially included in the study. Participants were aged between 8 and 11 years (mean age: 10 years and 1 month), and all were male. The study included only boys because all participants who met the inclusion criteria during the recruitment period were male. Although this reduced sample heterogeneity, it limits the generalizability of the findings to girls with ADHD.

Inclusion criteria required a previously established clinical diagnosis of ADHD. Information regarding diagnosis, medication use, physical activity participation, and other therapies was obtained from parents using a structured questionnaire prior to study enrollment. Based on parent-reported information, participants with known comorbid psychiatric or developmental conditions were not included. ADHD subtype distribution and symptom severity at diagnosis were not systematically recorded and were therefore not available for analysis.

The presence of specific learning difficulties, ongoing pharmacological treatment, and participation in organized extracurricular activities (e.g., football, basketball, or other sports) were not considered exclusion criteria. Among the 39 participants included in the final analyses, 7 were receiving pharmacological treatment and 32 were not. No significant differences in medication status were observed between the experimental and control groups. Information regarding medication dosage changes during the intervention period was not collected. Participants were not involved in other forms of ADHD treatment (e.g., psychotherapy or neurofeedback/biofeedback therapy) during the study period.

**Figure 1:**
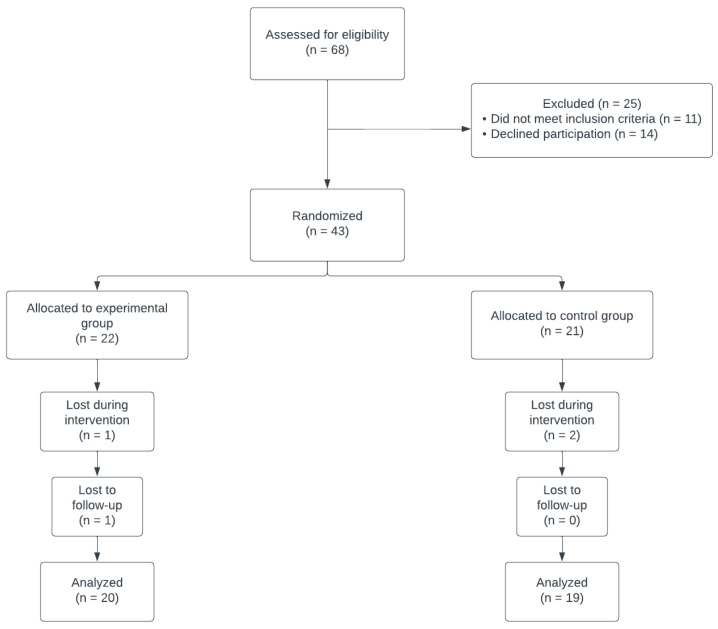
CONSORT flow diagram of participant recruitment, randomization, follow-up, and analysis.

A CONSORT flow diagram illustrating participant recruitment, allocation, follow-up, and inclusion in the final analyses is presented in Figure 1.

Sample Size and Statistical Power: ADHD symptom severity was considered the primary outcome of the study. To ensure adequate statistical power (0.80; α = 0.05; β = 0.20), an a priori sample size estimation was performed based on effect estimates reported in previous intervention studies examining ADHD symptoms in children. For the expected effect of physical activity on ADHD symptoms, the calculation was based on a change of 2.86 points on the Behavior Rating Scale reported by Ahmed and Mohamed [70], resulting in a minimum required sample of 14 participants. For the expected effect of mindfulness-based intervention on ADHD symptoms, the calculation was based on a change of 3.8 points on the Conners Parent Rating Scales reported by Santonastaso et al. [71], resulting in a minimum required sample of 34 participants. Therefore, a minimum sample size of 34 participants was considered necessary for the present study. A total of 43 participants were randomized, and 39 completed all study assessments.

Procedure and Data Analysis: During the intervention, three participants were excluded due to non-compliance, aggressive behavior, or withdrawal from the program (one from the EG and two from the CG). An additional participant from the EG was excluded due to incomplete assessment data. The final sample consisted of 39 participants (EG: *n* = 20; CG: *n* = 19). Data were analyzed using a per-protocol (PP) approach, including only participants who completed the intervention and all planned assessments. This approach allowed for a more accurate estimation of the intervention effects.

Instruments: Behavioral, emotional, and social problems were assessed using the ASEBA rating scale for children and adolescents [72,73], which evaluates children’s strengths, adaptive functioning, and behavioral, emotional, and social difficulties. In this study, the Teacher’s Report Form (TRF/6–18) was used for teacher ratings [74]. ADHD symptoms were measured using the ASEBA DSM-5–oriented ADHD subscale, which includes 13 items. The final score is expressed as a T-score reflecting the level of ADHD symptoms. The severity levels are classified as “normal” (T = 50–64), “borderline clinical” (T = 65–69), and “clinical” (T ≥ 70). Higher scores indicate greater adaptive difficulties and more pronounced ADHD symptoms. Perceptual-motor abilities were assessed using the Perceptual-Motor Skills Test [75,76], which consists of 31 tasks across seven dimensions: auditory perception of verbal instructions, auditory-language integration, visual discrimination of shapes and symbols, visual perception of forms and body positions, visuomotor coordination, rapid acquisition of visuomotor skills, and visuomotor integration. The test enables assessment of school readiness, identification of developmental deficits, and determination of strengths and weaknesses. Based on the results, children with developmental delays may be recommended for delayed school entry, individualized intervention programs, or inclusion in special education programs. In the present study, the test was used to objectively assess perceptual-motor functioning and monitor changes in attention, concentration, visuomotor integration, and cognitive-motor performance. The total score across all seven dimensions was used for analysis, with higher scores indicating better performance. Assessments were conducted by a psychologist according to the test manual, and administration lasted 60–90 min per child. Static balance, defined as the ability to maintain a stable body posture at rest by Panjan and Šarabon [77], was measured using a Kistler 9260AA6 force plate (Kistler, Winterthur, Switzerland) and Kistler MARS software (2022). Measurements were conducted at the Institute of Sport, Faculty of Sport, University of Ljubljana. All measurements were performed under standardized conditions (adequate lighting, absence of distractions, and constant temperature) [78,79]. Participants stood barefoot with both feet on the force plate in a natural upright posture, looking at a fixed point 3 m away at eye level [80], breathing normally, with hands placed on their hips. Each trial lasted 30 s, with three repetitions and a 3 min rest interval between trials. Data analysis was based on the sway path, representing the total displacement of the center of pressure in millimeters. The mean of three trials was calculated, where shorter path length indicates better static balance and longer path length indicates poorer balance. Dynamic balance, defined as the ability to maintain stability during movement, was assessed using the Biodex Balance System SD (Biodex Inc., Shirley, New York, NY, USA). The system measures movement along three axes (x, y, z) and calculates three stability indices: medial-lateral stability index (MLSI), anterior–posterior stability index (APSI), and overall stability index (OSI), which is the sum of MLSI and APSI [81]. The postural stability test was conducted at stability level 8. Each participant performed three trials lasting 30 s, with 10 s rest intervals. Prior to testing, participants’ height and weight were entered into the system. Participants stood barefoot in a relaxed posture, with foot position recorded to ensure consistency across trials. A familiarization trial was performed before testing. The platform moved in all directions, and deviations from a predefined zero point were recorded. Higher values indicate poorer dynamic balance. Data analysis was based on OSI, with the mean of three trials used for statistical analysis.

All assessment instruments used in this study are standardized measures with previously established psychometric properties, including satisfactory reliability and internal consistency reported in prior validation studies.

Procedure: All measurements were conducted at four time points: before the intervention, immediately after the intervention (three months after the first measurement), three months after the intervention, and six months after the intervention.

A comprehensive intervention program integrating physical activity and hypnosis-based mindfulness was implemented in the experimental group (EG) and was specifically designed for children with attention-deficit/hyperactivity disorder (ADHD). The intervention lasted 12 weeks and comprised two training sessions per week (24 sessions in total), each lasting 60 min and equally divided between physical activity and hypnosis-based mindfulness practice. The program was structured according to the FITT principle (Frequency, Intensity, Time, Type), with exercise intensity set at a moderate level and adapted to the developmental characteristics of children with ADHD. The physical activity component was based on fundamental movement patterns (e.g., walking, running, jumping, crawling), enriched with simple games and tasks incorporating additional cognitive demands, such as following complex instructions and performing dual-task activities. Particular emphasis was placed on the development of coordination, balance, visuomotor integration, auditory perception, and attention. The program followed a progressive structure, moving from individual activities to pair work and subsequently to group-based activities, thereby systematically promoting the development of social skills. In parallel, structured hypnosis-based mindfulness exercises were incorporated, focusing on increasing awareness of bodily sensations, thoughts, and emotions, as well as enhancing self-regulation and sustained attention.

An active control group (CG) was also included in the study design. This group participated in an emotion education program designed to control for non-specific intervention effects and to provide an active comparison condition. The control program likewise lasted 12 weeks (24 sessions, twice weekly, 60 min per session). Its content was based on reading emotion-related stories and engaging in guided discussions aimed at fostering the recognition, understanding, and awareness of emotions. The control condition did not include physical activity or hypnosis-based mindfulness components, allowing for a more precise attribution of observed effects to the specific elements of the experimental intervention. Based on these theoretical considerations and the objectives of the study, a systematic and carefully designed program of physical activity and hypnosis-based mindfulness was developed, grounded in the specific characteristics of children with ADHD and aligned with a holistic approach to their functioning.

The program targeted the development of visuomotor integration, coordination, balance, comprehension of auditorily presented verbal instructions, and awareness of one’s own body, thoughts, and emotions. The inclusion of dual-task activities (e.g., maintaining balance while simultaneously solving a cognitive task), simple games, fundamental movement patterns, and thematic units (e.g., animal-based activities: a giraffe, a turtle, a seal, etc.) was intended to simultaneously enhance cognitive and motor abilities while maintaining attention through play-based engagement. The training was progressively structured, beginning with individual task execution, followed by pair-based activities, and culminating in group-based performance. This approach facilitated the gradual development of social skills, reduced sensory overload, and provided a safe and supportive learning environment. Additionally, systematic hypnosis-based mindfulness exercises were incorporated to teach children to recognize their thoughts, feelings, and impulses, thereby strengthening their capacity for self-control, emotional regulation, and attentional focus. This additional program was termed hypnosis-based mindfulness because the mindfulness exercises were structured around techniques commonly used in clinical hypnosis, including relaxation as an induction process, focused attention, guided imagery, and therapeutic suggestions. The program was designed to comprehensively enhance both motor and cognitive abilities, which are commonly affected in children with ADHD.

The hypnosis-based mindfulness component was delivered as a structured, child-friendly procedure integrated into each intervention session. Each session followed a consistent sequence consisting of an introductory mindful warm-up, a central physical activity component with embedded mindfulness and hypnosis-based cues, and a concluding mindful relaxation phase. This structure was used to provide predictability, support attention, and increase replicability across sessions.

The introductory mindful warm-up lasted approximately 10–15 min. Children were allowed to perform the exercises either sitting or lying down, depending on comfort, and they could keep their eyes open or closed. Sufficient space was provided between participants to minimize distraction and physical contact. Each warm-up began with slow breathing and a brief relaxation/induction procedure in which children were guided to focus on inhalation and exhalation, shift attention away from external distractions, and prepare for the session. Child-friendly guided imagery was then introduced through animal-based themes. The first four themes in each cycle focused primarily on body and movement awareness, while the following four themes focused on thoughts and emotions. Across the 24 sessions, the thematic sequence included turtle, giraffe, lion, swallow, seal, frog, bear, and rabbit themes, which were repeated with increasing complexity. During the body- and movement-awareness exercises, children were guided to notice sensations such as contact between the feet or hands and the floor, muscle tension and relaxation, body position, balance, movement speed, breathing, and changes in posture. These exercises were organized around the turtle, giraffe, lion, and swallow themes. In the turtle-themed exercises, children practiced slow and quiet movement while focusing on the feet, the contact between the feet and the floor, and the sensation of moving slowly and deliberately. The giraffe-themed exercises emphasized upright posture, stretching, controlled body rotation, walking on the toes and heels, and awareness of body sway during balance control. The lion-themed exercises focused on bodily activation and release, including awareness of the hands and fingers, facial and oral movements, deep breathing, vocal exhalation, and subsequent relaxation. The swallow-themed exercises emphasized awareness of arm movements, posture, breathing, coordinated “wing-like” movements, and balance during movement. Together, these exercises were intended to help children direct attention to bodily sensations and movement in a concrete, playful, and developmentally appropriate way. During the thoughts-and-emotions exercises, children were encouraged to observe thoughts and feelings without immediate reaction. These exercises were organized around the seal, frog, bear, and rabbit themes. In the seal-themed exercise, children practiced observing thoughts by imagining them as clouds that could be noticed and gently allowed to move away. In the frog-themed exercise, they used guided imagery to imagine creative ideas and observe their thoughts. In the bear-themed exercise, they were encouraged to choose a positive intention for the day, while in the rabbit-themed exercise, they practiced silently directing kind thoughts toward others. These exercises were designed to support awareness of thoughts and emotions, emotional regulation, impulse control, and non-reactive self-observation.

The central part of each session consisted of approximately 30 min of adapted physical activity. This component included natural forms of movement, such as walking, running, crawling, creeping, and jumping, as well as elementary games and dual-task activities. Mindfulness and hypnosis-based cues were embedded into these activities by encouraging children to remain aware of bodily sensations, breathing, balance, movement, instructions, thoughts, and emotions during task performance. The tasks targeted visuomotor coordination, auditory perception of verbal instructions, eye–hand and eye–foot coordination, vestibular stimulation, balance, attention, and cognitive-motor integration. The intervention progressed from individual task performance during sessions 1–8, to pair-based activities during sessions 9–16, and group-based activities during sessions 17–24. This progression was intended to gradually increase cognitive, motor, and social demands while maintaining a safe and supportive environment.

The concluding mindful relaxation phase lasted approximately 10–15 min and focused on breathing, calming, and integration of the session experience. Children were again allowed to sit or lie comfortably with eyes open or closed. The relaxation exercises used concrete and age-appropriate imagery, such as blowing soap bubbles, cooling hot chocolate, breathing gently toward a candle flame, counting to five, inflating a balloon, blowing a bad mood away, bear breathing, and smelling a flower. These exercises emphasized slow inhalation, prolonged exhalation, relaxation, and the experience of calmness. Brief therapeutic suggestions were included to reinforce calm breathing, self-control, attention, confidence, and the ability to use these exercises outside the training setting, such as at home or at school.

All procedures were adapted to the developmental level of children with ADHD and were delivered in a playful, concrete, and non-threatening format.

All intervention sessions in both the experimental and active control groups were delivered by the same intervention leader, who held a Master’s degree in Kinesiology from the Faculty of Sport, University of Ljubljana, and had completed a diploma in Propedeutics as well as all academic requirements of the specialist postgraduate program in Systemic Psychotherapy at Sigmund Freud University Vienna, Ljubljana. To ensure intervention fidelity, all sessions followed predefined written protocols with standardized content, progression, duration, and thematic structure.

Statistical Analysis: Statistical analyses were conducted using IBM SPSS Statistics 25 (SPSS Inc., Armonk, NY, USA). Descriptive statistics included frequencies for categorical variables and means with standard deviations for continuous variables. The assumption of normality was assessed using the Shapiro–Wilk test for the total sample and separately for the experimental and active control groups. Homogeneity of variances was evaluated using Levene’s test, and sphericity was assessed using Mauchly’s test.

Baseline differences between groups were examined prior to the intervention. For outcome variables with no significant baseline differences, intervention effects were analyzed using a two-way repeated-measures analysis of variance (ANOVA), with time (baseline, post-intervention, 3-month follow-up, and 6-month follow-up) as the within-subject factor and group (experimental vs. active control) as the between-subject factor. For variables showing significant baseline differences between groups (i.e., static balance), a two-way repeated-measures analysis of covariance (ANCOVA) was used to control for baseline differences.

The main effects of time and the time × group interaction were examined for all outcome measures. Results are presented as mean differences, 95% confidence intervals, and *p*-values. Post hoc pairwise comparisons between measurement time points were adjusted using the Bonferroni correction to control for multiple testing. Given 12 pairwise comparisons, the adjusted significance threshold was set at *p* < 0.0042 (0.05/12). Only comparisons meeting this adjusted threshold were considered statistically significant and marked accordingly in the tables.

A per-protocol (PP) approach was used for data analysis. Only participants who completed the intervention and all scheduled assessments were included in the final analyses. This approach was selected to evaluate intervention effects among participants who received the full intervention and provided complete outcome data.

In addition, Spearman’s rank correlation coefficient was used to examine associations between changes in balance performance and changes in ADHD symptom severity. Statistical significance was set at *p* < 0.05 for all analyses unless otherwise specified.

## 3. Results

Baseline characteristics of the participants are presented in Table 1. No statistically significant differences were observed between the experimental and active control groups regarding age, medication status, physical activity participation, or study location at baseline, indicating that the groups were comparable prior to the intervention.

Table 2 presents differences in teacher-rated ADHD symptom severity (TRF/6–18) between measurement points in the experimental and active control groups. *p*-values were adjusted using the Bonferroni correction for multiple comparisons, with statistical significance set at *p* < 0.0042.

Hypothesis 1 predicted that children in the experimental group would demonstrate greater reductions in ADHD symptom severity at post-intervention and follow-up assessments compared with children in the control group.

Repeated-measures analysis revealed a significant time × group interaction (Pillai’s Trace = 0.883, F(3,35) = 88.287, *p* < 0.001, partial η^2^ = 0.883), indicating a very large intervention effect.

As shown in Table 2, statistically significant reductions in teacher-rated ADHD symptom severity were observed in the experimental group between baseline and post-intervention (mean difference = 16.4, *p* < 0.001), baseline and 3-month follow-up (mean difference = 15.8, *p* < 0.001), and baseline and 6-month follow-up (mean difference = 9.7, *p* < 0.001). No statistically significant changes were observed in the control group at any measurement point.

These findings support Hypothesis 1 and indicate that the intervention was associated with both short-term and longer-term reductions in ADHD symptom severity.

**Figure 2:**
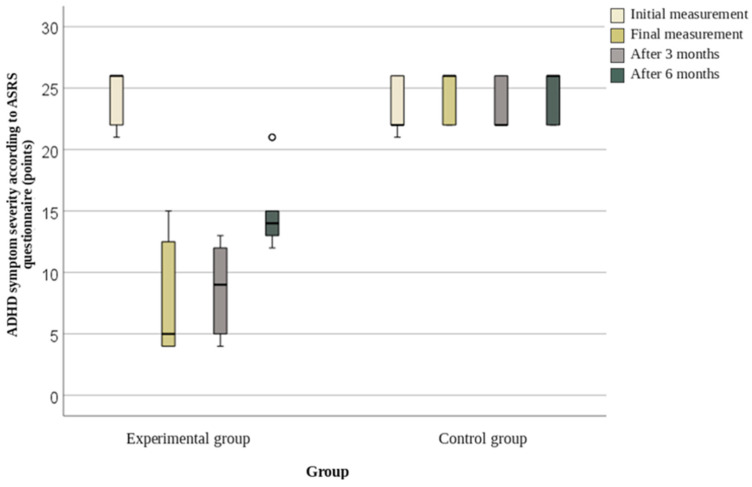
Effect of the intervention on the severity of ADHD symptoms in participants based on teacher ASRS (TRF/6–18; [74]) across different measurement points. Note: ASRS (scores)—expression of ADHD symptoms in children using the ASEBA TRF/6–18 questionnaire ([74]), presented in scores. The figure shows the expression of ADHD symptoms in the participants using a quantile plot, where the lower line represents the minimum, the upper line the maximum, the lower edge of the box the first quartile, the middle line the median (second quartile), and the upper edge the third quartile. The circle indicates outlying values (outliers).

Figure 2 illustrates changes in ADHD symptom severity across measurement points. A marked reduction was observed in the experimental group following the intervention and was largely maintained during follow-up, whereas symptom severity remained relatively stable in the control group.

Table 3 presents differences in perceptual–motor abilities between measurement points in the experimental and active control groups. *p*-values were adjusted using the Bon-ferroni correction for multiple comparisons, with statistical significance set at *p* < 0.0042.

Hypothesis 2 predicted that children in the experimental group would demonstrate greater improvements in perceptual–motor abilities at post-intervention and follow-up assessments compared with children in the control group.

Repeated-measures analysis revealed a significant time × group interaction for perceptual–motor abilities (Pillai’s Trace = 0.565, F(3,35) = 15.157, *p* < 0.001, partial η^2^ = 0.565), indicating a large intervention effect.

As shown in Table 3, statistically significant improvements in perceptual–motor abilities were observed in the experimental group between baseline and post-intervention (mean difference = −26.8, *p* < 0.001), baseline and 3-month follow-up (mean difference = −27.4, *p* < 0.001), and baseline and 6-month follow-up (mean difference = −22.8, *p* < 0.001). No comparable improvements were observed in the control group.

These findings support Hypothesis 2 and indicate that the intervention was associated with both short-term and longer-term improvements in perceptual–motor abilities.

**Figure 3:**
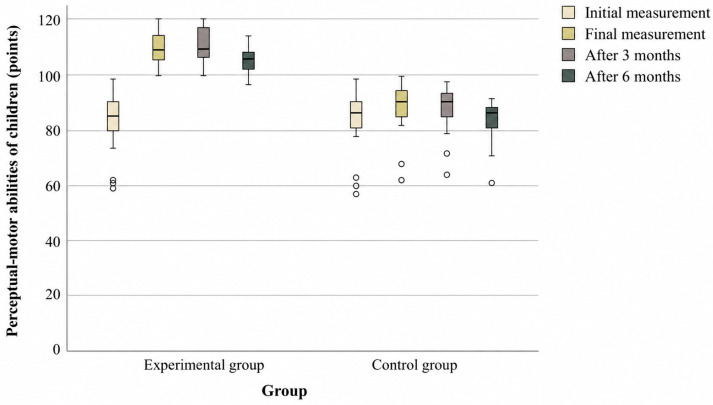
Effect of the intervention on perceptual-motor abilities based on ratings obtained using the assessment scale by Mitić Petek [75,76] across different measurement points. Note. Perceptual-motor abilities (scores)—results of perceptual-motor abilities based on ratings obtained using the ZMS Test (Mitić Petek [75,76]), expressed in points. The figure presents participants’ scores using a quantile plot, where the lower line represents the minimum, the upper line the maximum, the lower edge of the box the first quartile, the middle line the median (second quartile), and the upper edge the third quartile. Circles indicate outlying values (outliers).

Figure 3 illustrates changes in perceptual–motor abilities across measurement points. A marked improvement was observed in the experimental group following the intervention and was maintained at both follow-up assessments, whereas scores in the control group remained relatively stable over time.

Table 4 presents differences in static balance results between measurement points in the experimental and active control groups. *p*-values were adjusted using the Bonferroni correction for multiple comparisons, with statistical significance set at *p* < 0.0042.

Hypothesis 3 predicted that children in the experimental group would demonstrate greater improvements in static balance at post-intervention and follow-up assessments compared with children in the control group.

Repeated-measures analysis revealed a significant time × group interaction for static balance (Pillai’s Trace = 0.610, F(3,35) = 18.257, *p* < 0.001, partial η^2^ = 0.610), indicating a large intervention effect.

As shown in Table 4, a statistically significant improvement in static balance was observed in the experimental group between baseline and post-intervention measurements (mean difference = 239.1 mm, *p* < 0.001). However, no statistically significant differences were observed between baseline and the 3-month or 6-month follow-up assessments. In the control group, a significant difference was also observed between baseline and post-intervention measurements (mean difference = −170.2 mm, *p* = 0.001), whereas no significant changes were detected at follow-up assessments. A significant baseline difference between groups was observed; therefore, results were analyzed using repeated-measures ANCOVA.

These findings partially support Hypothesis 3, indicating a short-term improvement in static balance following the intervention, but no clear evidence of sustained long-term effects.

**Figure 4:**
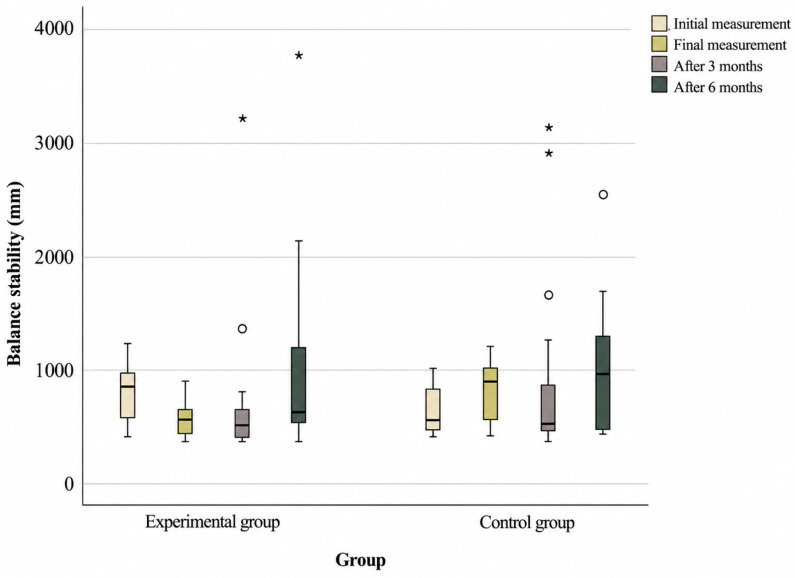
Effect of the intervention on static balance outcomes across different measurement points. Note: The results of static equilibrium are expressed in mm. In the figure, we have shown the results for the test specimens using a quantile plot, where the lower line represents the minimum, the upper line the maximum, the lower edge of the rectangle the first quartile, the middle line the median (second quartile), and the upper edge the third quartile. Circles and stars indicate outlier values.

Figure 4 illustrates changes in static balance across measurement points. An improvement was observed in the experimental group immediately after the intervention, whereas follow-up measurements suggested a reduction in this effect over time.

Table 5 presents differences in dynamic balance results between measurement points in the experimental and active control groups. *p*-values were adjusted using the Bonferroni correction for multiple comparisons, with statistical significance set at *p* < 0.0042.

Hypothesis 4 predicted that children in the experimental group would demonstrate greater improvements in dynamic balance at post-intervention and follow-up assessments compared with children in the control group.

Repeated-measures analysis revealed a significant time × group interaction for dynamic balance (Pillai’s Trace = 0.904, F(3,35) = 109.387, *p* < 0.001, partial η^2^ = 0.904), indicating a very large intervention effect.

As shown in Table 5, statistically significant improvements in dynamic balance were observed in the experimental group between baseline and post-intervention (mean difference = 2.527, *p* < 0.001), baseline and 3-month follow-up (mean difference = 2.423, *p* < 0.001), and baseline and 6-month follow-up (mean difference = 1.959, *p* < 0.001). In contrast, no significant improvements were observed in the control group.

These findings support Hypothesis 4 and indicate that the intervention was associated with both short-term and longer-term improvements in dynamic balance.

**Figure 5:**
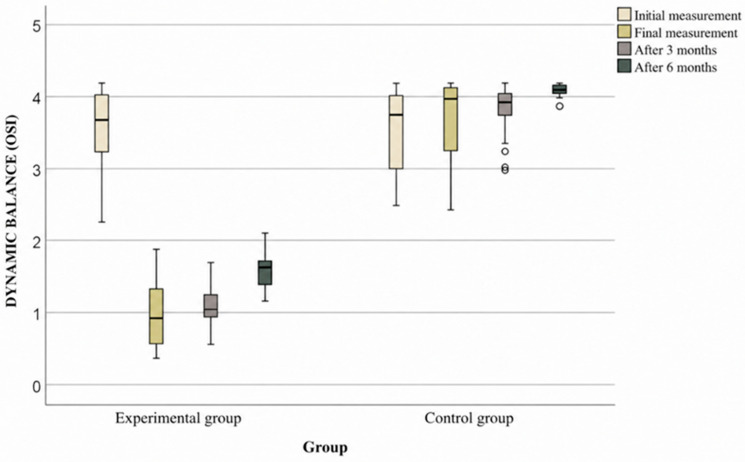
Effect of the intervention on dynamic balance results across different measurement points. Note: Dynamic balance results expressed using the OSI index. The figure presents the results for participants using a quantile plot, where the lower line represents the minimum, the upper line represents the maximum, the lower edge of the box represents the first quartile, the middle line represents the median (second quartile), and the upper edge represents the third quartile. Circles indicate outlier values.

Figure 5 illustrates changes in dynamic balance across measurement points. A marked improvement was observed in the experimental group following the intervention and was maintained at both follow-up assessments, whereas no comparable improvement was observed in the control group.

## 4. Discussion

The present findings may be interpreted through several complementary mechanisms. First, the reduction in ADHD symptom severity observed in the experimental group is consistent with previous studies suggesting that structured physical activity may improve attention, behavioral inhibition, executive functioning, and emotional regulation in children with ADHD [55,56,57,58,59,60,61,62]. Physical activity may contribute to these effects through increased arousal regulation, enhanced dopaminergic and noradrenergic activity, and improved modulation of serotonin and endogenous opioids, all of which are relevant to attention, motivation, mood, and impulse control [49,54]. In the present intervention, physical activity was not limited to general exercise but included structured tasks requiring sustained attention, inhibition, following verbal instructions, visuomotor coordination, and dual-task performance. These characteristics may have provided repeated practice of cognitive control in a motivating, play-based motor context.

Second, the hypnosis-based mindfulness component may have contributed to symptom reduction by strengthening self-regulation, focused attention, emotional awareness, and the ability to monitor bodily sensations, thoughts, and impulses. Previous mindfulness-based studies in children and adolescents with ADHD have reported improvements in attention, working memory, executive task performance, cognitive control, behavioral regulation, and impulsivity [64,65,66,67]. In our study, mindfulness exercises were adapted within a clinical hypnosis-informed framework, including relaxation, focused attention, guided imagery, and therapeutic suggestions. These elements may have helped children enter a calmer and more focused state, improve awareness of internal experiences, and practice more adaptive responses to impulsive or emotionally dysregulated behavior. This may partly explain the sustained reduction in teacher-rated ADHD symptoms observed after the intervention.

Third, the improvements in perceptual-motor abilities may be related to the specific motor and cognitive demands embedded in the program. The intervention included activities targeting balance, coordination, visuomotor integration, auditory perception of verbal instructions, body awareness, and progressive social interaction through individual, pair-based, and group activities. These components overlap with the domains assessed by the Perceptual-Motor Skills Test and may therefore have directly trained the abilities that improved after the intervention.

Fourth, the improvements in dynamic balance and the short-term improvement in static balance may be explained by repeated exposure to balance-related tasks, dual-task activities, and exercises requiring postural control under attentional demands. Balance control depends on the integration of visual, vestibular, proprioceptive, somatosensory, musculoskeletal, and central nervous system processes, and it is also influenced by attention and executive control. Because children with ADHD frequently show difficulties in motor coordination, postural control, and executive functioning, a program that simultaneously trains movement, attention, and self-regulation may be particularly relevant for this population. The stronger and more sustained effect observed for dynamic balance may reflect the fact that the intervention emphasized active movement, coordination, and balance during play-based tasks, whereas static balance may require more continuous or targeted practice to maintain long-term gains.

Overall, our results are in line with previous research showing beneficial effects of physical activity on ADHD symptoms, executive functioning, attention, and motor skills [55,56,57,58,59,60,61,62], as well as studies reporting positive effects of mindfulness-based interventions on attention, working memory, cognitive control, behavioral regulation, and impulsivity [64,65,66,67]. However, the present study extends this literature by examining an integrated intervention combining physical activity with hypnosis-based mindfulness and by including objective measures of static and dynamic balance in addition to behavioral and perceptual-motor outcomes. Nevertheless, the mechanisms proposed here remain interpretative, as the present study did not directly measure neurophysiological markers, emotional regulation, or specific neuropsychological mechanisms such as inhibitory control, working memory, or sustained attention. Future studies should include objective measures of executive functioning, autonomic regulation, neuropsychological performance, and physical activity intensity to better clarify how such combined interventions produce their effects.

A few limitations of the present study should be acknowledged. First, although the final sample size met the minimum requirements estimated from previous intervention studies, the overall sample was relatively small (*n* = 39), limiting statistical power and generalizability. In addition, all participants were boys aged 8–11 years; therefore, the findings cannot be directly generalized to girls with ADHD or to children outside this age range. Future studies should include larger, more diverse, and gender-balanced samples. The findings should therefore be interpreted as preliminary evidence supporting the potential usefulness of this intervention in boys with ADHD aged 8–11 years.

Second, the experimental intervention combined physical activity and hypnosis-based mindfulness, whereas the active control group received emotion education. Consequently, the independent effects of the two intervention components cannot be determined. Future studies should use multi-arm designs to examine the individual and combined contributions of physical activity and hypnosis-based mindfulness.

A further limitation is that analyses were conducted using a per-protocol approach, which may increase susceptibility to attrition bias and potentially overestimate treatment effects compared with an intention-to-treat analysis. By including only participants who completed the intervention and all planned assessments, the observed effects may reflect treatment efficacy under optimal adherence conditions rather than effectiveness in routine clinical settings. However, the dropout rate in the present study was relatively low (4/43; 9.3%), which may partially reduce this bias. Future studies should consider applying intention-to-treat procedures to provide more conservative and generalizable estimates of intervention effectiveness.

Additional limitations include the absence of full blinding and the reliance on teacher-reported ADHD symptoms. Although participants and their parents were not informed of the specific study hypotheses or group allocation, the intervention leader was aware of group assignment, which may have introduced performance bias. Furthermore, teacher ratings may be influenced by subjective perceptions and observer bias.

## 5. Conclusions

Attention-deficit/hyperactivity disorder (ADHD) is a neurodevelopmental condition characterized by impairments in attention, hyperactivity, and impulsivity that substantially affect children’s daily functioning. In addition to cognitive and behavioral difficulties, many children with ADHD experience challenges in motor coordination, balance, and perceptual–motor functioning.

The findings of the present study suggest that a structured intervention combining physical activity and hypnosis-based mindfulness was associated with reductions in ADHD symptom severity and improvements in perceptual–motor abilities, static balance, and dynamic balance in boys with ADHD aged 8–11 years. Improvements in ADHD symptoms, perceptual–motor abilities, and dynamic balance were maintained during the follow-up period, suggesting the potential durability of some intervention effects. However, long-term improvements in static balance were not observed, indicating that continued practice may be necessary to maintain gains in this domain.

The results should be interpreted with caution given the relatively small sample size, the inclusion of boys only, and the multicomponent nature of the intervention, which does not allow the independent effects of physical activity and hypnosis-based mindfulness to be determined. Furthermore, the use of a per-protocol analytical approach and the absence of full blinding should be considered when interpreting the findings.

Overall, the findings suggest that the integration of physical activity and hypnosis-based mindfulness may represent a promising and practically feasible complementary approach for supporting children with ADHD. Nevertheless, larger and methodologically rigorous randomized controlled trials are needed to confirm these findings, examine the individual contributions of each intervention component, and clarify their long-term effectiveness.

## Figures and Tables

**Table 1:** Baseline characteristics of participants.

Characteristic	Experimental Group (n = 20)	Control Group (n = 19)	*p*
Age (years), mean ± SD	10.00 ± 0.56	10.16 ± 0.60	0.403
Medication use, n (%)			1.000
•Receiving ADHD medication	4 (20.0%)	3 (15.8%)	
•Not receiving ADHD medication	16 (80.0%)	16 (84.2%)	
Physical activity participation, n (%)			0.688
•Never	15 (75.0%)	13 (68.4%)	
•Once per week	3 (15.0%)	2 (10.5%)	
•Twice per week	2 (10.0%)	4 (21.1%)	
Study location, n (%)			1.000
•Ljubljana	13 (65.0%)	13 (68.4%)	
•Maribor	7 (35.0%)	6 (31.6%)	

Note: Values are presented as mean ± standard deviation (SD) or number (percentage). Group differences were examined using the Mann–Whitney U test (age) and Fisher’s exact test (categorical variables).

**Table 2:** Differences in ADHD symptom severity scores (TRF/6–18) between time points in experimental and active control groups.

Group	Comparison	Mean Diff (I–J)	95% CI	*p*
EG	Baseline vs. post	16.4 *	14.0–18.7	<0.001
	Baseline vs. 3 month	15.8 *	13.5–18.1	<0.001
	Baseline vs. 6 month	9.7 *	7.9–11.5	<0.001
	Post vs. 3 month	−0.6	−3.0–1.9	1.000
	Post vs. 6 month	−6.7 *	−8.9–−4.4	<0.001
	3 vs. 6 month	−6.1 *	−8.5–−3.7	<0.001
CG	Baseline vs. post	−1.2	−3.6–1.3	1.000
	Baseline vs. 3 month	−0.5	−2.9–1.9	1.000
	Baseline vs. 6 month	−1.0	−2.8–0.9	1.000
	Post vs. 3 month	0.6	−1.9–3.1	1.000
	Post vs. 6 month	0.2	−2.1–2.5	1.000
	3 vs. 6 month	−0.4	−2.9–2.1	1.000

Note. I–J—mean differences between ratings of ADHD symptom severity scores obtained using the Teacher’s Report Form (TRF/6-18) by Achenbach System of Empirically Based Assessment [74]; 95% CI—95% confidence interval; *p*—statistical significance calculated using a two-factor repeated measures analysis of variance; baseline measurements—assessments conducted before the start of the intervention; post-intervention measurements—assessments conducted after completion of the intervention; measurements after three months—assessments conducted three months after the end of the intervention; measurements after six months—assessments conducted six months after the end of the intervention. The table presents *p*-values adjusted using the Bonferroni correction for multiple testing. * *p* < 0.0042.

**Table 3:** Differences in perceptual-motor abilities based on ratings obtained using the Perceptual-Motor Skills Test assessment scale by Mitić Petek [75,76] between time points in experimental and active control groups.

Group	Comparison	Mean Diff (I–J)	95% CI	*p*
EG	Baseline vs. post	−26.8 *	−33.3, −20.3	<0.001
	Baseline vs. 3 month	−27.4 *	−33.7, −21.0	<0.001
	Baseline vs. 6 month	−22.8 *	−29.4, −16.2	<0.001
	Post vs. 3 month	−0.6	−2.2, 1.1	1.000
	Post vs. 6 month	4.0	0.9, 7.1	0.0057
	3 vs. 6 month	4.6 *	2.2, 6.9	<0.001
CG	Baseline vs. post	−4.8	−11.5, 1.9	0.316
	Baseline vs. 3 month	−4.8	−11.3, 1.7	0.269
	Baseline vs. 6 month	−0.4	−7.2, 6.3	1.000
	Post vs. 3 month	−0.1	−1.7, 1.6	1.000
	Post vs. 6 month	4.4 *	1.2, 7.6	0.0029
	3 vs. 6 month	4.4 *	2.0, 6.8	<0.001

Note. I–J—mean differences between ratings of perceptual-motor abilities obtained using the assessment scale by Mitić Petek [75,76]; 95% CI—95% confidence interval; *p*—statistical significance calculated using a two-factor repeated measures analysis of variance; baseline measurements—assessments conducted before the start of the intervention; post-intervention measurements—as-sessments conducted after completion of the intervention; measurements after three months—as-sessments conducted three months after the end of the intervention; measurements after six months—assessments conducted six months after the end of the intervention. The table presents *p*-values adjusted using the Bonferroni correction for multiple testing. * *p* < 0.0042.

**Table 4:** Differences in static balance results between time points in experimental and active control groups.

Group	Comparison	Mean Diff (I–J)	95% CI	*p*
EG	Baseline vs. post	239.1 *	127.8 to 350.4	<0.001 *
	Baseline vs. 3 month	111.4	−324.5 to 547.3	1.000
	Baseline vs. 6 month	−185.8	−600.2 to 228.7	1.000
	Post vs. 3 month	−127.7	−530.7 to 275.4	1.000
	Post vs. 6 month	−424.9	−808.7 to −41.1	0.023
	3 vs. 6 month	−297.2	−816.0 to 221.7	0.713
CG	Baseline vs. post	−170.2 *	−284.4 to −55.9	0.0011 *
	Baseline vs. 3 month	−241.7	−688.9 to 205.5	0.843
	Baseline vs. 6 month	−352.1	−777.3 to 73.2	0.160
	Post vs. 3 month	−71.5	−485.0 to 342.0	1.000
	Post vs. 6 month	−181.9	−575.7 to 211.9	1.000
	3 vs. 6 month	−110.4	−642.7 to 422.0	1.000

Note. I–J—mean differences in static balance results measured on a force platform (center of pressure displacement path in millimeters; mean of three trials); 95% CI—95% confidence interval; *p*—statistical significance calculated using a two-factor repeated measures analysis of covariance; baseline measurements—assessments conducted before the start of the intervention; post-intervention measurements—assessments conducted after completion of the intervention; 3-month follow-up measurements—assessments conducted three months after the end of the intervention; 6-month follow-up measurements—assessments conducted six months after the end of the intervention. The table presents *p*-values adjusted using the Bonferroni correction for multiple testing. * *p* < 0.0042.

**Table 5:** Differences in dynamic balance results between time points in experimental and active control groups.

Group	Comparison	Mean Diff (I–J)	95% CI	*p*
EG	Baseline vs. post	2.527 *	2.223–2.832	<0.001
	Baseline vs. 3 month	2.423 *	2.071–2.775	<0.001
	Baseline vs. 6 month	1.959 *	1.610–2.309	<0.001
	Post vs. 3 month	−0.104	−0.343–0.134	1.000
	Post vs. 6 month	−0.568 *	−0.890–−0.246	<0.001
	3 vs. 6 month	−0.464 *	−0.666–−0.261	<0.001
CG	Baseline vs. post	−0.205	−0.518–0.107	0.450
	Baseline vs. 3 month	−0.290	−0.651–0.071	0.188
	Baseline vs. 6 month	−0.584 *	−0.942–−0.225	<0.001
	Post vs. 3 month	−0.085	−0.329–0.160	1.000
	Post vs. 6 month	−0.378	−0.709–−0.048	0.017
	3 vs. 6 month	−0.294 *	−0.502–−0.086	0.002

Note: I–J—mean differences between dynamic balance results measured using the Biodex Balance System (OSI index; average of three measurements); 95% CI—95% confidence interval; *p*—statistical significance calculated using a two-way repeated-measures ANOVA; baseline measurements—measurements taken before the start of the intervention; post-intervention measurements—measurements taken after the completion of the intervention; three-month follow-up measurements—measurements taken three months after the end of the intervention; six-month follow-up measurements—measurements taken six months after the end of the intervention. The table presents values adjusted using the Bonferroni correction for multiple comparisons. * *p* < 0.0042.

## Data Availability

The data used in this study are available from the corresponding author upon reasonable request.

## Abbreviations

The following abbreviations are used in this manuscript:

ADHD	Attention-Deficit/Hyperactivity Disorder
QoL	Quality of Life
EG	Experimental Group
CG	Control Group
PA	Physical activity
HBMT	Hypnosis-based Mindfulness training
ASEBA TRF/6–18	Achenbach System of Empirically Based Assessment-Teacher’s Report Form
ANOVA	Analysis of Variance
ANCOVA	Analysis of Covariance
NCCIH	National Center for Complementary and Integrative Health
DSM	Diagnostic and Statistical Manual of Mental Disorders
